# Association between triglyceride glucose index and subclinical left ventricular systolic dysfunction in patients with type 2 diabetes

**DOI:** 10.1186/s12944-023-01796-1

**Published:** 2023-03-08

**Authors:** Yanyan Chen, Jianfang Fu, Yi Wang, Ying Zhang, Min Shi, Cheng Wang, Mengying Li, Li Wang, Xiangyang Liu, Shengjun Ta, Liwen Liu, Zeping Li, Xiaomiao Li, Jie Zhou

**Affiliations:** 1 Department of Endocrinology, Xijing Hospital, Air Force Medical University, 710032 Xi’an, Shaanxi, China; 2 Department of Ultrasound, Xijing Hospital, Air Force Medical University, Xi’an, Shannxi 710032 China; 3 Nanchang University Queen Mary School, Nanchang, 330038 China

**Keywords:** Type 2 diabetes mellitus, Triglyceride glucose index, Global longitudinal strain, Left ventricular systolic function, Insulin resistance

## Abstract

**Background:**

The triglyceride glucose (TyG) index has been considered a new biomarker for the diagnosis of angiocardiopathy and insulin resistance. However, the association of the TyG index with subclinical left ventricular (LV) systolic dysfunction still lacks comprehensive exploration. This study was carried out to examine this relationship in patients with type 2 diabetes mellitus (T2DM).

**Methods:**

A total of 150 T2DM patients with preserved LV ejection fraction (LVEF ≥ 50%) from June 2021 to December 2021 were included in this study. The subclinical LV function was evaluated through global longitudinal strain (GLS), with the predefined GLS < 18% as the cutoff for subclinical LV systolic dysfunction. The TyG index calculation was obtained according to ln (fasting triglycerides (mg/dL) × fasting glucose (mg/dL)/2), which was then stratified into quartiles (TyG index—Q).

**Results:**

Analyses of clinical characteristics in the four TyG indexes-Q (Q1 (TyG index ≤ 8.89) *n* = 38, Q2 (8.89 < TyG index ≤ 9.44) *n* = 37, Q3 (9.44 < TyG index ≤ 9.83) *n* = 38, and Q4 (TyG index > 9.83) *n* = 37) were conducted. A negative correlation of the TyG index with GLS (*r* = -0.307, *P* < 0.001) was revealed according to correlation analysis. After gender and age were adjusted in multimodel logistic regression analysis, the higher TyG index (OR 6.86; 95% CI 2.44 to 19.30; *P* < 0.001, Q4 vs Q1) showed a significant association with GLS < 18%, which was still maintained after further adjustment for related clinical confounding factors (OR 5.23, 95% CI 1.12 to 24.51, *p* = 0.036, Q4 vs Q1). Receiver operator characteristic analysis indicated a diagnostic capacity of the TyG index for GLS < 18% (area under curve: 0.678;* P* < 0.001).

**Conclusions:**

A higher TyG index had a significant association with subclinical LV systolic dysfunction in T2DM patients with preserved ejection fraction, and the TyG index may have the potential to exert predictive value for myocardial damage.

## Background

A causal relationship exists between diabetes mellitus and heart failure. For instance, diabetic cardiomyopathy as a diffuse cardiomyopathy resulting from glucose metabolism disorder has received increasing interest in recent years, which generally manifests as pathological cardiac remodeling and systolic and diastolic dysfunction and may eventually develop into overt heart failure [1, 2]. Studies have demonstrated insulin resistance and/or hyperinsulinemia as the source of the cascade that contributes to diabetic cardiomyopathy [3, 4]. The early stages of diabetic cardiomyopathy are usually ignored and underestimated in clinics. Some studies have shown the existence of asymptomatic systolic LV dysfunction in patients with type 2 diabetes mellitus (T2DM) with hidden cardiac disease manifestations [5] and suggest the emergence of reduced global longitudinal strain (GLS) at an early stage of this disorder process prior to the detectability of ejection fraction (EF) transformations [6]. Therefore, early identification followed by punctual intervention is of vital significance for individuals at high risk of diabetic cardiomyopathy. The TyG index is indicated to serve as an alternative biomarker of insulin resistance, which is convenient and reliable [7–9] and is closely related to cardiovascular disease [10]. However, there is not yet enough evidence to evaluate the clinical value of the TyG index on subclinical LV dysfunction in diabetes. Accordingly, our objective was to explore the correlation of the TyG index with LV longitudinal systolic function in T2DM patients without heart disease.

## Methods

### Study subjects

A total of 165 hospitalized patients with T2DM categorized by World Health Organization criteria [11] at the Department of Endocrinology of Xijing Hospital of Air Force Medical University during the period of June 2021 to December 2021 were enrolled. The exclusion criteria were as follows: (1) LVEF < 50%; (2) moderate-to-severe aortic/mitral valve stenosis or insufficiency; (3) a coronary artery disease history or other heart disease; (4) arrhythmia such as left bundle-branch block, frequent ventricular premature complexes or atrial fibrillation; and (5) too poor speck tracking image quality for analysis. According to the exclusion criteria, 15 participants were excluded. Ultimately, 150 patients with T2DM were included in the present study.

### Data collection

Demographic data were provided by the electronic medical record system, with diabetes duration, gender, age, systolic and diastolic pressures, and medication covered. All patients were measured for height and weight through specially assigned personnel on the admission day, with body mass index (BMI) defined as weight/height^2^ (kg/m^2^). Hypertension was described as systolic blood pressure (SBP) ≥ 140 mmHg and/or diastolic blood pressure (DBP) ≥ 90 mmHg without antihypertensive medication or with a previous hypertension physician identification. After an at least 8-h overnight fast, an evaluation of total cholesterol, triglycerides, high- and low-density lipoprotein cholesterol, and uric acid was performed with an automatic biochemical analyzer. The fasting glucose index was detected with glycosylated hemoglobin (HbA1c) checked by high-performance liquid chromatography. Immunoturbidimetry depending on a COBAS INTEGRA 400 plus autoanalyzer (Germany) was carried out to determine the urinary albumin-to-creatinine ratio (UACR). The ln[fasting triglycerides (mg/dL) × fasting glucose (mg/dL)/2] [7] was used to identify the TyG index, and the patients were grouped into Q1 (TyG index ≤ 8.89), Q2 (8.89 < TyG index ≤ 9.44), Q3 (9.44 < TyG index ≤ 9.83), and Q4 (TyG index > 9.83) based on the TyG index levels.

### Conventional echocardiography

The ultrasound measurements of all subjects were collected according to the guidelines of the American Society of Echocardiography [12]. Two-dimensional (2D) echocardiography (Philips Healthcare, iE33 system, X5-1 probe) was performed on each subject accompanied by electrocardiogram. LV fractional shortening (LVFS), LVEF, heart rate, and stroke volume of each subject were analyzed. Afterwards, the pulse Doppler sampling volume was located under the mitral valve to perform the measurement on the early diastolic blood flow velocity (E peak) and late diastole (A peak) to calculate the E/A ratio, then to measure the early diastolic mitral annulus motion velocity (E' peak) to calculate the E/E' ratio by placing it at the septal side of the mitral annulus.

### Speck-tracking echocardiography

**Fig. 1:**
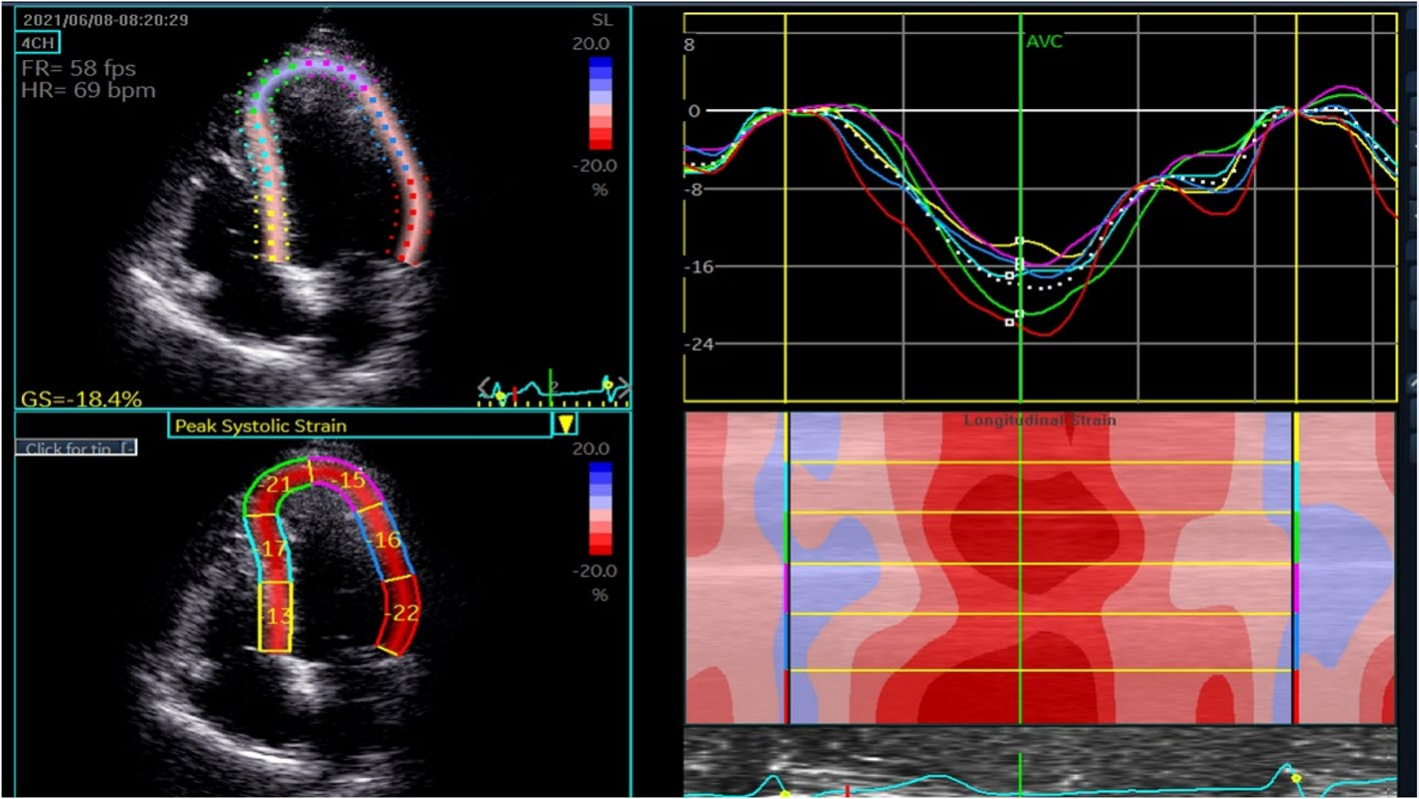
Acquisition of left ventricular apical four-chamber strain imaging by two-dimensional speckle tracking echocardiography in type 2 diabetic patients

Echocardiography images for 2D speck-tracking echocardiography (STE) with a rate of 60–90 frames/s in three cardiac cycles were acquired. Then, the LV views of the apical four-chamber, apical two-chamber and apical three-chamber were analyzed using QLAB 8.1 2D imaging system. The trace of the endocardial border at end-diastole was performed in a manual manner to obtain the 2D strain‒time curve and bull's-eye plot of 17 segments of LV (Fig. 1). The trace was adjusted based on a visual assessment of the tracking quality by observer, and the untraceable images of spots elicited by atrial fibrillation were excluded from the subsequent analysis. The LV GLS for the average value of the three peak strains in systole was calculated for subclinical LV systolic function evaluation. Based on previous studies and the latest guidelines of the European Association of Cardiovascular Imaging, the predefined cutoff of GLS < 18% was adopted to evaluate subclinical LV systolic dysfunction [12–14].

### Statistical analyses

A Kolmogorov‒Smirnov test was performed to test the normality of data, and a nonparametric test was implemented where data did not meet a normal distribution. Data satisfying normal distribution were expressed as the means ± SDs, and conversely, as median and interquartile range. Categorical variables were described in the form of percentages n (%). One-way ANOVA or the Kruskal‒Wallis test was used to test for differences between groups. The Bonferroni test was adopted for post hoc comparisons. Least Pearson's Chi-square test was performed on categorical variable comparisons. The correlation between the TyG index and GLS was evaluated according to Pearson correlation coefficients. Three forced-entry logistic regression models were performed to determine the independent association of GLS < 18% with TyG index: model 1 (an unadjusted model), mode 2 (a multivariable model) adjusted for age and gender, and model 3 (a multivariable model) adjusted for age, gender, diabetes duration, systolic pressure, HbA1c, BMI, hypertension, heart rate, logarithmic microalbuminuria, LVEF and insulin therapy. To confirm an alternative index for identifying reduced GLS < 18%, models covering the TyG index and HbA1c were established, followed by a comparison of both according to the area under the receiver operating characteristic (ROC) curves (AUC). The measurement of GLS was performed by one professional physician to avoid interobserver and intraobserver variability. To prevent the ambiguity of negative size to a value, the GLS was given in the absolute value form. All statistical analyses were carried out on IBM SPSS statistics 26.0, with a *P* value < 0.05 considered statistically significant.

## Results

### Clinical characteristics

A total of 150 T2DM patients (mean age: 53.4 ± 13.8 years, diabetes duration: 10.25 ± 7.22 years, male: 96 (64.0%)) were included, with the clinical features across quartiles of the TyG index listed in Table 1, which indicated an increased hypertension prevalence in subjects with a higher TyG index, accompanied by higher levels of systolic pressure, fasting glucose, HbA1c, heart rate, triglycerides, low-density lipoprotein cholesterol, and total cholesterol (all *P* < 0.05). Moreover, insulin therapy statistically varied across the TyG index quartiles (*P* = 0.025) without obvious differences in sex, age, diabetes duration or BMI among groups (*P* > 0.05).

**Table 1:** Clinical characteristics of participants by quartiles of TyG index

Characteristic	Q1 (TyG index ≤ 8.89) *n* = 38	Q2 (8.89 < TyG index ≤ 9.44) *n* = 37	Q3 (9.44 < TyG index ≤ 9.83) *n* = 38	Q4 (TyG index > 9.83) *n* = 37	*P value*
Male, n (%)	23 (60.5)	26 (70.3)	26 (68.4)	21 (56.8)	0.573
Age, years	52.5 ± 15.3	55.8 ± 13.3	52.6 ± 11.2	53.1 ± 15.3	0.692
BMI, kg/m^2^	23.29 ± 3.88	23.87 ± 3.54	24.82 ± 3.10	24.51 ± 4.11	0.273
Diabetes duration, years	10.79 ± 7.08	8.57 ± 6.97	9.17 ± 6.78	12.29 ± 7.79	0.115
Heart rate, bpm	72.45 ± 8.73	73.65 ± 12.12	77.74 ± 2.71	81.31 ± 4.02^ce^	**0.008**
SBP, mmHg	127.08 ± 15.29	135.08 ± 14.53	131.26 ± 14.62	139.59 ± 24.44^c^	**0.019**
DBP, mmHg	76.16 ± 9.30	78.97 ± 9.53	77.39 ± 9.20	80.43 ± 14.86	0.359
HbA1c, (%)	7.93 ± 1.64	8.49 ± 1.84	9.05 ± 1.96	9.75 ± 2.46^ce^	**0.001**
FPG, mmol/l	8.15 (6.38–9.40)	10.00 (7.85–14.45)^a^	11.50(10.10–13.73)^b^	14.50(10.25–18.15)^ce^	**< 0.001**
Total cholesterol, mmol/l	3.57 ± 1.05	3.72 ± 1.10	4.07 ± 0.90	4.83 ± 1.57^cef^	**< 0.001**
HDL-C, mmol/l	1.19 ± 0.32	1.10 ± 0.53	1.09 ± 0.47	1.03 ± 0.36	0.426
LDL-C, mmol/l	1.95 ± 0.91	2.17 ± 0.97	2.61 ± 1.27^b^	2.76 ± 1.22^ce^	**0.006**
Triglyceride, mmol/l	0.70 (0.61–0.99)	1.16 (0.87–1.45)^a^	1.58 (1.29–1.97) ^bd^	2.77 (2.13–3.81)^cef^	**< 0.001**
Apolipoproteins A1, g/l	1.27 ± 0.22	1.14 ± 0.18^a^	1.16 ± 0.18	1.23 ± 0.21	**0.028**
Apolipoproteins B, g/l	0.56 ± 0.17	0.63 ± 0.21	0.81 ± 0.49^b^	0.82 ± 0.31^c^	**0.001**
Albuminuria, mg/l	11.30 (8.13–15.18)	12.85 (8.83–20.08)	9.20 (8.00–15.00)	14.90 (8.30–105.00)	0.109
UACR, mg/mmol	1.45 (0.77–2.42)	1.45 (1.03–3.55)	1.70 (0.75–2.73)	2.41(1.12–16.47)	0.172
Uric acid, umol/l	301.43 ± 66.61	315.00 ± 80.30	327.30 ± 87.99	341.92 ± 88.45	0.177
hypertension, n (%)	8 (21.1)	20 (54.1)^a^	19 (50.0)^b^	21(56.8)^c^	**0.006**
Diabetic nephropathy, n (%)	5 (13.2)	8 (21.6)	8 (21.1)	14 (37.8)	0.081
Diabetic retinopathy, n (%)	3 (7.9)	3 (8.1)	5 (13.2)	10 (27.0)	0.058
Medical treatment					
ACEI/ARB, n (%)	4 (10.5)	8 (21.6)	11 (28.9)	11 (29.7)	0.163
CCB, n (%)	5 (13.2)	10 (27.0)	8 (21.1)	8 (21.6)	0.523
Statin, n (%)	8 (21.1)	7 (18.9)	10 (26.3)	10 (27.0)	0.805
Insulin, n (%)	23 (60.5)	12 (32.4)^a^	24 (63.2)	22 (59.5)	**0.025**
SGLT-2I, n (%)	4 (10.5)	3 (8.1)	4 (10.5)	3 (8.1)	0.968
GLP-1RA, n (%)	5 (13.2)	2 (5.4)	4 (10.5)	4 (10.8)	0.723
Metformin, n (%)	27 (71.1)	26 (70.3)	27 (71.1)	25 (67.6)	0.986
αGI, n (%)	15 (39.5)	13 (35.1)	12 (31.6)	13 (35.1)	0.914

Values are presented as the mean ± SD, median (interquartile range) or n (%). Bold indicates values of *P* < 0.05

Abbreviations: *TyG* Triglyceride glucose, *BMI* Body mass index, *SBP* Systolic blood pressure, *DBP* Diastolic blood pressure, *FPG* Fasting plasma glucose, *TC* Total cholesterol, *HDL-C* High-density lipoprotein cholesterol, *LDL-C* Low-density lipoprotein cholesterol, *UACR* Urinary albumin-to-creatinine ratio, *CCB* Calcium channel blocker, *ACEI* Angiotensin-converting enzyme inhibitor, *ARB* Angiotensin II receptor blocker, *SGLT-2I* Sodium glucose cotransporter 2 inhibitor, *GLP-1RA* Glucagon-like peptide-1 receptor agonist, *α-GI* α-glucosidase inhibitor. The Bonferroni test was adopted for post hoc comparisons

^a^ *P* < 0.05 between the 1st quartile and 2nd quartile

^b^ *P* < 0.05 between the 1st quartile and 3rd quartile

^c^ *P* < 0.05 between the 1st quartile and 4th quartile

^d^ *P* < 0.05 between the 2nd quartile and 3rd quartile

^e^ *P* < 0.05 between the 2nd quartile and 4th quartile

^f^ *P* < 0.05 between the 3rd quartile and 4th quartile

### Association of the TyG index with GLS

Table 2 displays the characteristics of LV function stratified by quartiles of the TyG index. No statistically significant differences were observed in traditional echocardiographic parameters, that is, LVEF, LVFS, stroke volume, E, E', E/A and E/E' across TyG index quartiles (all *P* > 0.05). However, the GLS exhibited a stepwise decrease in line with the increase in TyG index quartile (19.23 ± 3.28 vs 18.87 ± 2.79 vs 17.22 ± 3.56 vs 16.67 ± 3.31, *P* = 0.001). Pearson correlation analysis indicated strong and negative correlations of the TyG index (*r* = -0.307, *P* < 0.001) and HbA1c (*r* = -0.470, *P* < 0.001) with GLS.

**Table 2:** Characteristics of LV function stratified by quartiles of the TyG index

Echocardiographic indexes	Q1 (TyG index ≤ 8.89) *n* = 38	Q2 (8.89 < TyG index ≤ 9.44) *n* = 37	Q3 (9.44 < TyG index ≤ 9.83) *n* = 38	Q4 (TyG index > 9.83) *n* = 37	*P value*
LV EF, %	60.14 ± 3.89	60.19 ± 5.55	60.13 ± 5.01	59.91 ± 4.30	0.995
LV FS, %	31.94 ± 3.36	32.09 ± 4.34	32.41 ± 3.92	31.73 ± 3.86	0.912
Stroke volume, ml	47.22 ± 8.59	48.47 ± 9.64	48.22 ± 9.91	45.61 ± 7.50	0.561
E,cm/s	67.12 ± 19.01	67.90 ± 14.12	65.97 ± 13.74	64.55 ± 15.03	0.850
A,cm/s	73.48 ± 19.17	80.40 ± 21.18	75.07 ± 19.81	83.26 ± 19.40	0.181
E',cm/s	7.92 ± 3.01	7.01 ± 2.41	7.74 ± 2.75	6.77 ± 2.44	0.303
E/A ratio	1.00 ± 0.38	1.36 ± 2.82	0.94 ± 0.36	0.83 ± 0.39	0.508
E/E' ratio	10.36 ± 4.23	10.22 ± 3.49	9.52 ± 3.01	10.14 ± 3.11	0.825
GLS, %	19.23 ± 3.28	18.87 ± 2.79	17.22 ± 3.56^a^	16.67 ± 3.31^bc^	**0.001**

Bold indicates *P* < 0.05

Abbreviations: *TyG* Triglyceride glucose, *LVEF* Left ventricular ejection fraction, *LVFS*, *LV* fractional shortening, *E*Peak early diastolic mitral flow velocity, *A* Peak late diastolic mitral flow velocity, *E/A* Peak early diastolic (E-wave) and late diastolic (A-wave) velocity ratio, *E/E'* Mitral inflow E and mitral E' annular velocity ratio, *GLS* Global longitudinal strain. The Bonferroni test was adopted for post hoc comparisons

^a^ *P* < 0.05 between the 1st quartile and 3rd quartile

^b^ *P* < 0.05 between the 1st quartile and 4th quartile

^c^ *P* < 0.05 between the 2nd quartile and 4th quartile

Table 3 displays the logistic regression results based on the TyG index quartile. Taking Q1 as the reference, the risks in the Q3 and Q4 groups of GLS < 18% were found to be significantly higher in comparison to the Q1 group in the univariate model (Q3 vs Q1: OR 4.29, 95% CI 1.63 to 11.35, *P* = 0.003; Q4 vs Q1: OR 5.83, 95% CI 2.15 to 15.82, *P* < 0.001, respectively). After gender and age adjustment, the relation of the TyG index of the 3rd quartile and 4th quartile with GLS < 18% still existed (Q3 vs Q1: OR 4.87, 95% CI 1.78 to 13.28, *P* = *0*.002; Q4 vs Q1: OR 6.86, 95% CI 2.44 to 19.30, *P* < 0.001, respectively). With further adjustment for confounders of age, gender, diabetes duration, systolic pressure, HbA1c, BMI, hypertension, heart rate, logarithmic microalbuminuria, LVEF and insulin therapy, the higher quartile of the TyG index remained an independent risk indicator related to GLS < 18% (Q3 vs Q1: OR 4.52, 95% CI 1.12 to 18.27, *P* = 0.034; Q4 vs Q1: OR 5.23, 95% CI 1.12 to 24.51, *P* = 0.036).

**Table 3:** Logistic regression analysis of GLS < 18% by TyG index quartiles

	**Model 1**		**Model 2**		**Model 3**	
	OR (95%CI)	*P value*	OR (95%CI)	*P value*	OR (95%CI)	*P value*
Q1 (TyG index ≤ 8.89)	Reference	—	Reference	—	Reference	—
Q2 (8.89 < TyG index ≤ 9.44)	1.34 (0.50, 3.64)	0.561	1.59 (0.57, 4.48)	0.380	1.28 (0.30, 5.55)	0.741
Q3 (9.44 < TyG index ≤ 9.83)	4.29 (1.63, 11.35)	**0.003**	4.87 (1.78, 13.28)	**0.002**	4.52 (1.12, 18.27)	**0.034**
Q4 (TyG index > 9.83)	5.83 (2.15, 15.82)	**< 0.001**	6.86 (2.44, 19.30)	**< 0.001**	5.23 (1.12, 24.51)	**0.036**

Bold indicates *P* < 0.05. Abbreviations: TyG, triglyceride glucose; OR, odds ratio; CI, confidential interval. Model 1: unadjusted; Model 2: adjusted for age and gender; Model 3: further adjusted for age, gender, diabetes duration, systolic pressure, HbA1c, BMI, hypertension, heart rate, logarithmic microalbuminuria, LVEF and insulin therapy

**Fig. 2:**
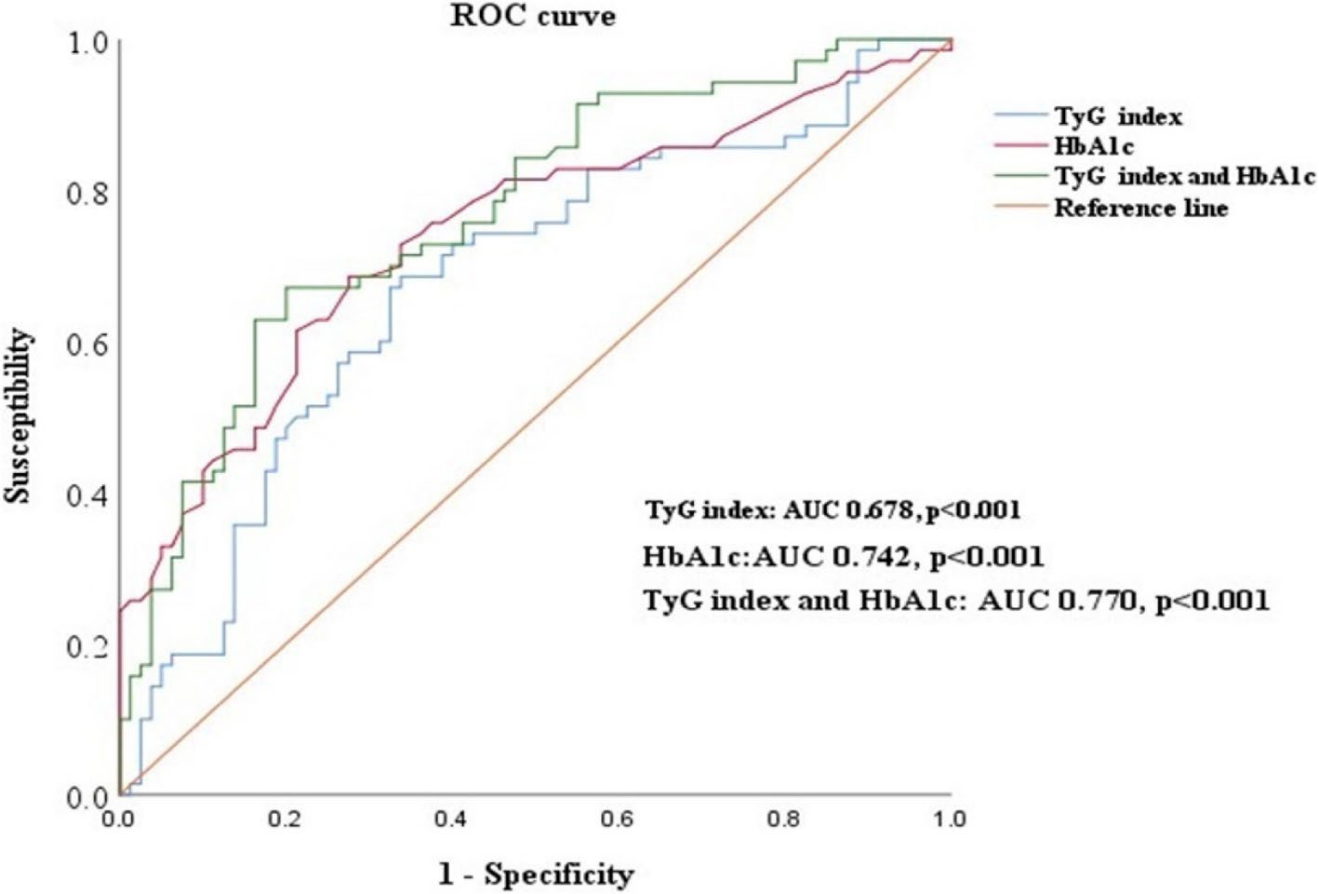
Receiver‑operating characteristic curves for the prediction of reduced GLS (< 18%) in patients with type 2 diabetes using the TyG index and HbA1c. Abbreviations: GLS: global longitudinal strain; HbA1c: glycosylated hemoglobin

The ROC curves depicted in Fig. 2 demonstrate the diagnostic validity of the TyG index in identifying subclinical LV systolic dysfunction (GLS < 18%). Notably, the TyG index with a cutoff value of 9.6 (AUC: 0.678; *P* < 0.001) displayed a sensitivity of 73.8% with a specificity of 54.3% for predicting GLS < 18%. HbA1c also exhibited a high AUC of 0.742, a sensitivity of 62.9% and a specificity of 76.2% for reduced GLS < 18% (*P* < 0.001). Subsequently, the composite variable with the TyG index and HbA1c combined showed increased AUC and diagnostic values (AUC: 0.770; sensitivity: 65.7%, specificity: 80.0%, *P* < 0.001).

**Fig. 3:**
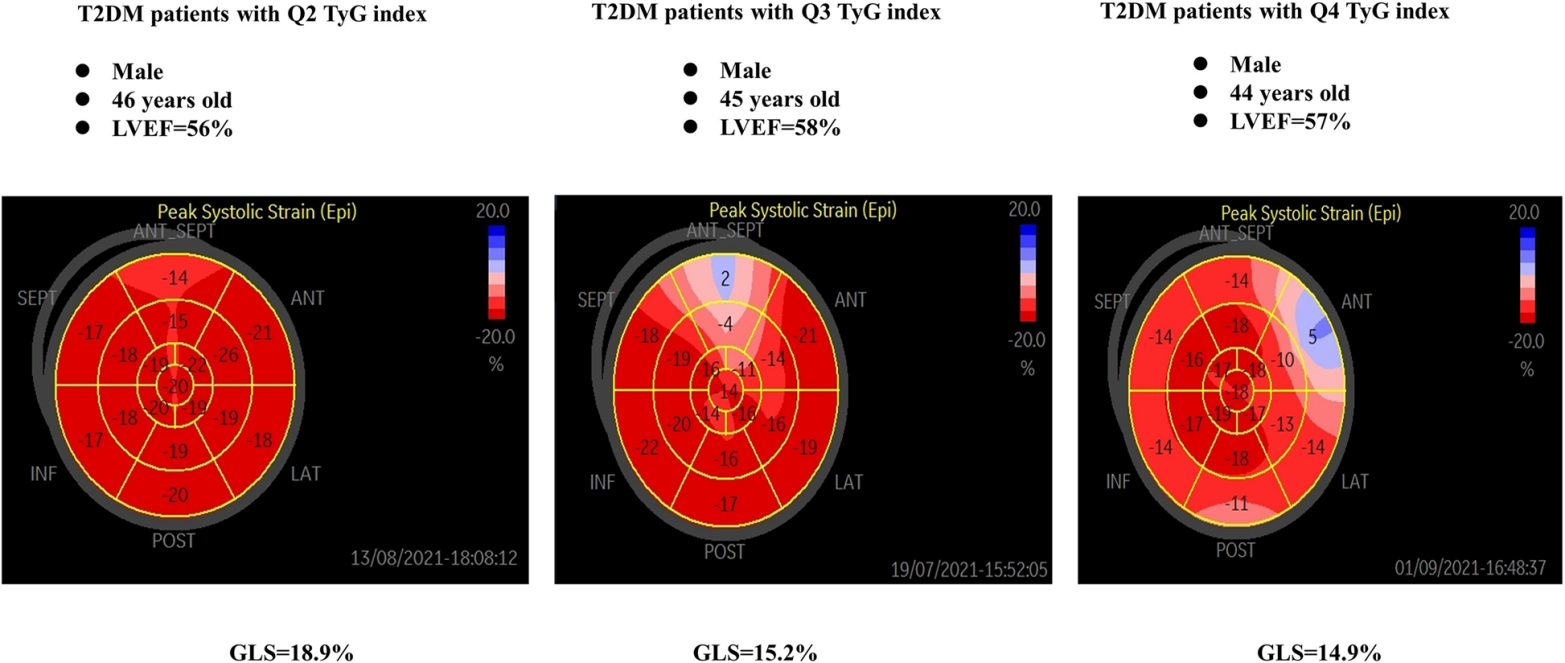
Three bull’s eye plots of representative cases with reduced GLS with high TyG index quartiles (Q2〜Q4). Abbreviations: GLS: global longitudinal strain; Q: quartile

Figure 3 shows three bull’s eye plots of representative cases with reduced GLS with high TyG index quartiles (Q2〜Q4).

## Discussion

To the best of our knowledge, the present study was the first to explore the association of the TyG index with LV longitudinal myocardial function in patients with type 2 diabetes and preserved ejection fraction. The results demonstrated a close relationship of the increased TyG index with an elevated risk of LV longitudinal myocardial dysfunction. Moreover, it was found that the composite parameters of the TyG index and HbA1c exhibited a certain value for the identification of reduced GLS < 18%.

The correlation between heart failure and diabetes has been notably confirmed by epidemiological and clinical studies [15–17]. However, among diabetes-related complications, diabetic cardiomyopathy, as an "entity", remains poorly understood. An increasing number of studies have indicated a hidden subclinical period in diabetic cardiomyopathy, featuring subtle abnormalities in function and structure [18]. In this context, asymptomatic LV dysfunction, defined as abnormal diastolic or systolic function without clinically detectable heart disease, is frequently reported in T2DM patients, which is expected to be between 50 and 70% [19] and presented as LV systolic dysfunction in one-third of patients [20]. Recently, GLS has been adopted as a preferred indicator to evaluate global LV systolic function, considering the longitudinal subendocardial fibers as the most vulnerable fibers that are first damaged by metabolic disorders in the early stage of diabetic heart disease [21, 22]. Ernande et al. also proposed the presence of LV longitudinal dysfunction in T2DM patients with preserved LVEF but normal LV diastolic function, which was defined as GLS < 18% [23]. The specific mechanism of this disorder remains to be uncovered. Metabolic characterizations have indicated that impaired insulin metabolic signaling is a contributing pathophysiological abnormality associated with diabetic cardiomyopathy [3, 4].

To date, the standard estimation of insulin resistance, the hyperinsulinemic-euglycemic clamp (HEGC) test, still requires diagnostic technology, which is expensive and is not available for basic-level hospital utilization. The TyG index, as an ideal surrogate of insulin resistance regardless of insulin treatment status, has been widely validated to be robustly related to cardiovascular events [24–26]. However, evidence on the validity of the TyG index on LV longitudinal myocardial function in those without prominent symptoms of heart failure is still not sufficient. Indeed, individuals with insulin resistance tend to develop systematic metabolic disorders, including dyslipidemia, hyperglycemia, and hypertension, which were also reported by the present study, as the highest quartile of the TyG index tended to be associated with a high prevalence of hypertension, poor blood glucose control and lipid levels. These interactions will significantly promote insulin resistance. Notably, these patients were more prone to suffering from reduced GLS than those in the lowest quartile. Subsequently, the multimodel logistics regression analysis demonstrated the independent association of a higher TyG index (ORs: 4.52 and 5.23 in the Q3 and Q4 groups compared with the Q1 group) with subclinical LV systolic dysfunction assessed by GLS < 18%. This result was consistent with the view of Ikonomidis et al., who reported that insulin resistance was related to GLS and resulted in LV longitudinal dysfunction in the immediate family of T2DM patients [27]. In fact, reduced coronary flow reserve has been shown to be a crucial determinant of LV longitudinal subendocardial myocardial fiber deformation. In addition, insulin resistance may induce myocardial injury through various other mechanisms, including oxidative stress, fibrosis, autonomic nervous dysfunction and inefficient energy metabolism [28, 29]. Accordingly, the TyG index may serve as the contributing reference for detecting cardiac involvement at a relatively earlier stage of diabetic heart disease.

In a study with a large cohort followed for 10 years, Sánchez-Íñigo et al. first proposed a positive correlation of the TyG index (AUC: 0.708) with heart events [30]. Similarly, the ROC curve plotted here indicated the clinical validity of the TyG index (AUC: 0.678) for reduced GLS. More interestingly, compared to HbA1c and the composite index, the TyG index with a cutoff value of 9.6 showed the highest sensitivity but the lowest specificity in predicting subclinical LV systolic dysfunction. This finding is in agreement with previous studies in which insulin resistance has been recognized as both a pathogenic trigger and a predictor of cardiovascular events [10]. Moreover, the AUC of the TyG index binding to HbA1c (AUC: 0.770) observed in this study provided an incremental predictive value for poor cardiac outcomes. Despite the absence of an absolute illustration of the underlying mechanisms of this relationship, it has been determined that the TyG index represents the combined effect of "glycotoxicity" and "lipid toxicity", which prominently contribute to the reduced endocardial collateral flow density and the impaired coronary microcirculation in patients with T2DM [4, 31]. Consequently, it is not unexpected to observe systemic lipid disturbances, including elevated total cholesterol, triglycerides, low-density lipoprotein cholesterol levels, and apolipoproteins in the present study, which in turn evoke oxidative stress and inflammation, with the potential to elicit lipotoxic cardiomyopathy [32]. These pathologies further support the triggering role of insulin resistance in the early initiation of myocardial function changes in diabetic patients, such as hyperglycemia and dysfunction of lipid oxidation and utilization [33].

### Study strengths and limitations

Previous studies have mostly focused on patients with existing symptoms of heart failure. In contrast, more emphasis was placed on early attention to the LV subclinical phase of T2DM patients in the present study. The relatively time-consuming requirement, high cost, and professional features of speckle tracking echocardiography and the accumulated training to perform effective measurement and analysis may limit its application in daily clinical practice for general diabetes physicians without enough experience with this technique. As stated above, the hyperglycemia elicited by insulin resistance activates the cascade of diabetic heart dysfunction, which induces metabolic disorders, followed by endothelial dysfunction, cardiac hypertrophy and fibrosis [34, 35]. HEGC has been confirmed to be closely related to poor prognosis in type 2 diabetes, exerting a praisable validity for the prevention and treatment of those patients. However, the complex operation and uneconomical test limit its wide availability in clinical practice. The homeostasis model assessment of insulin resistance (HOMA-IR) is considered another preferential index, which requires the measurement of fasting insulin. Because of the absence of a standardized method for insulin measurement or the recognized cutoff value, it is relatively difficult to apply in primary hospitals. Promisingly, the TyG index has been validated as a more accurate novel measurement of insulin resistance compared to HOMA⁃IR [36]. More prominently, this index is available and inexpensive in reality. The outcomes of this study demonstrated the validity of the TyG index to assist clinicians in screening people at high risk of cardiovascular events, exerting a more prominent role in the prevention and intervention of diabetic cardiomyopathy. Thus, it is recommended to perform punctual monitoring of the TyG index as soon as possible. For people in the T2DM population with a high TyG index in particular, metabolic disorders are suggested to be controlled earlier, and advanced hypoglycemic medicines for cardiovascular protection should be administered to effectively avoid and delay the occurrence and development of diabetic heart disease and ultimately bring clinical benefits to patients.

The limitations of this study should also be considered. First, as a result of the cross-sectional study, the specific causal relationship of the TyG index with reduced GLS remains unclear. Second, not all patients without coronary artery disease have undergone invasive coronary angiography. Third, despite the analysis on the medication of the patients, the underlying contributions of medications for lipid-lowering and LV function improvements failed to be controlled in this study. Fourth, insulin resistance parameters were not analyzed in this study. Last, covariates involved in the multivariable regression models were taken as potential confounders based on previous studies [37, 38] or their biological plausibility, which partly limits the further application of the research findings.

## Conclusions

In conclusion, the present study demonstrated a close relationship between an elevated TyG index and decreased GLS, which could be adopted as a sensitive and practical index to predict subclinical LV systolic dysfunction. Therefore, the TyG index should be punctually monitored for T2DM patients with preserved LVEF, which is expected to effectively identify the occurrence of diabetic heart disease and delay its development.

## Data Availability

The datasets analyzed during the current study are available from the corresponding author on reasonable request.

## Abbreviations

TyG: Triglyceride glucose
LVEF: Left ventricular ejection fraction
T2DM: Type 2 diabetes mellitus
GLS: Global longitudinal strain
Q: Quartile
BMI: Body mass index
SBP: Systolic blood pressure
DBP: Diastolic blood pressure
HbA1c: Glycosylated hemoglobin
UACR: Urinary albumin-to-creatinine ratio
LVFS: Left ventricular fractional shortening
E peak: Early diastolic blood flow velocity
A peak: Late diastolic blood flow velocity
E' peak: Early diastolic mitral annulus motion velocity
2D STE: Two-dimensional speck-tracking echocardiography
AUC: Area under the curve
ROC: Receiver operating characteristic
HEGC: Hyperinsulinemic-euglycemic clamp
HOMA-IR: Homeostasis model assessment of insulin resistance
